# Classification of the Multiple Stages of Parkinson’s Disease by a Deep Convolution Neural Network Based on ^99m^Tc-TRODAT-1 SPECT Images

**DOI:** 10.3390/molecules25204792

**Published:** 2020-10-19

**Authors:** Shih-Yen Hsu, Li-Ren Yeh, Tai-Been Chen, Wei-Chang Du, Yung-Hui Huang, Wen-Hung Twan, Ming-Chia Lin, Yun-Hsuan Hsu, Yi-Chen Wu, Huei-Yung Chen

**Affiliations:** 1 Department of Medical Imaging and Radiological Science, I-Shou University, No. 8, Yida Road., Jiao-su Village Yan-chao District, Kaohsiung City 82445, Taiwan; h.shihyen@gmail.com (S.-Y.H.); ed110880@edah.org.tw (L.-R.Y.); ctb@isu.edu.tw (T.-B.C.); yhhuang@isu.edu.tw (Y.-H.H.); 2 Department of Anesthesiology, E-DA Cancer Hospital, I-Shou University, No.1, Yida Road, Jiao-su Village, Yan-chao District, Kaohsiung City 82445, Taiwan; 3 Department of Information Engineering, I-Shou University, No.1, Sec. 1, Syuecheng Road., Dashu District, Kaohsiung 84001, Taiwan; wcdu@isu.edu.tw; 4 Department of Life Sciences, National Taitung University, No.369, Sec. 2, University Road, Taitung 95092, Taiwan; twan@nttu.edu.tw; 5 Department of Nuclear Medicine, E-DA Hospital, I-Shou University, No.1, Yida Rd, Jiao-su Village, Yan-chao District, Kaohsiung 82445, Taiwan; ed101186@edah.org.tw (M.-C.L.); edps900002@edah.org.tw (Y.-H.H.)

**Keywords:** SPECT, Parkinson’s disease, deep learning, convolution neural network

## Abstract

Single photon emission computed tomography (SPECT) has been employed to detect Parkinson’s disease (PD). However, analysis of the SPECT PD images was mostly based on the region of interest (ROI) approach. Due to limited size of the ROI, especially in the multi-stage classification of PD, this study utilizes deep learning methods to establish a multiple stages classification model of PD. In the retrospective study, the 99mTc-TRODAT-1 was used for brain SPECT imaging. A total of 202 cases were collected, and five slices were selected for analysis from each subject. The total number of images was thus 1010. According to the Hoehn and Yahr Scale standards, all the cases were divided into healthy, early, middle, late four stages, and HYS I~V six stages. Deep learning is compared with five convolutional neural networks (CNNs). The input images included grayscale and pseudo color of two types. The training and validation sets were 70% and 30%. The accuracy, recall, precision, F-score, and Kappa values were used to evaluate the models’ performance. The best accuracy of the models based on grayscale and color images in four and six stages were 0.83 (AlexNet), 0.85 (VGG), 0.78 (DenseNet) and 0.78 (DenseNet).

## 1. Introduction

In recent years, single photon emission computed tomography (SPECT) has been used to estimate tumor growth, genetic treatments, brain function detection and cardiovascular diseases [1,2]. It is a mature imaging tool. In nuclear imaging, γ-rays are emitted from radiopharmaceuticals and received by a gamma camera placed around the object. The signal was then passed through the internal components, including scintillation crystals, photomultiplier tubes, positioning circuits, pulse height analyzers, etc. Finally, a radionuclide species distribution is obtained by image reconstruction algorithms.

However, the imaging process of nuclear medicine images is deeply disturbed by scattering events (called Compton scattering), especially for single photon emission computed tomography, which can cause quantitative image bias, low contrast and affect image quality [3,4], which prevents the fixed image results from appearing correctly. For example, in Siemens’ Symbia^®^ S SPECT imaging the scattering ratio (under 172 keV energy) may reach 50% when using 111-In for trunk imaging (scatter fraction = total counts (172 keV)/total counts (247 keV)) [5]. This means one of every two signals from the camera is a self-scattering event, so the nuclear medicine image is blurred, but nuclear medicine imaging is mainly used to detect radioactive tracer’s spatial distribution in the human body. Moreover, it is particularly sensitive to detecting specific tissues or organs (such as the thyroid gland, brain, heart, liver, kidney, etc.), which is also called physiological imaging modality.

Parkinson’s disease (PD) is mainly caused by insufficient secretion of neurotransmitters (dopamine, acetylcholine) in the brain’s basal nucleus and neurodegeneration in the substantia nigra area. In past research, the dopamine neurons from the substantia nigra area are degraded due to increasing age or external factors [6]. This makes the presynaptic release of dopamine neurons insufficient and decreases dopamine transporter (DAT) levels. This then causes inhibition signals to be transmitted to the globus pallidus by the tail nucleus, and the putamen is reduced. Moreover, the inhibition signals to the ventral anterior thalamus are increased, which leads to insufficient signals to the premotor cortex and causes dyskinesia. Generally, almost 70% to 80% of the dopamine signals are lost by the time clinical signs begin, so early diagnosis of Parkinson’s disease is essential [7]. For the early stages of Parkinson’s disease or senior subjects, dopamine drugs (such as levodopa, dopamine agonists, anticholinergics) could be used to distinguish patients’ symptoms and pathological properties by the reaction after taking the drug. Furthermore, the use of non-invasive imaging diagnosis techniques (such as SPECT) is also an essential reference.

In earlier nuclear medicine ^123^I-β-CIT was used to detect dopamine transporters. Because of the drug’s uptake in the striatum, it had a significant correlation with the standard Unified Parkinson Disease Rating Scale (UPDRS—the comprehensive Parkinson’s disease rating scale is divided into four parts, namely patient psychology, daily living ability, motor function, and treatment complications) adopted to determine the PD stage [8]. However, the pharmacokinetics of ^123^I-β-CIT were very slow. It took up to 24 h to reach equilibrium after administering the drug, requiring more examination time. After that, other drugs were developed (such as ^99m^Tc-TRODAT-1, ^123^I-FP-CIT, ^123^I-altropane) [9,10,11,12]. Although the imaging time can be shortened, the probability of non-specific binding was increased. Furthermore, the cost of the medicines became more expensive, so they could not be routinely applied in the clinic until the advent of ^99m^Tc- TRODAT-1, which has high sensitivity and specificity for DaTscan. In this study, the PD stage was classified using the Hoehn and Yahr scale (HYS). According to the literature the HYS is highly correlated with UPDRS scores [13]. The following table lists information about the HYS scale (Table 1).

^99m^Tc-TRODAT-1 is a successful preclinical dopamine-labeled drug [14,15]. ^99m^Tc-TRODAT-1 could be used in SPECT brain imaging to evaluate a variety of neurological disorders in the brain (such as Parkinson’s disease, hemichorea-hemiballism, Tourette’s syndrome, multiple system atrophy, Wilson’s disease, depression, etc.) [16,17,18,19]. In addition ^99m^Tc-TRODAT-1 has good performance to detect dopamine transporters in the striatum [20,21]. In the past, most scientists used the region of interest (ROI) method to calculate and extract features for the analysis and classification of SPECT dopamine transporter scans and then utilized statistical classification methods or machine learning methods to analyze the results [22,23,24]. This kind of method was limited by the selected ROI size and the extraction value of the characteristic parameters. This affects the disease stage determination result. The deep convolutional neural network (CNN) method was applied to the image classification area [25,26] by feeding a whole image to the deep network. This can effectively solve the problems in determining the ROI.

This study aimed to explore deep learning algorithm performance for Parkinson disease (PD) classification by SPECT imaging using the ^99m^Tc-TRODAT-1 tracer method. Among deep learning algorithms, five different convolutional neural network architectures such as AlexNet, GoogLeNet, Residual Neural Network, VGG, and DenseNet were utilized to classify and model Parkinson’s disease stages. It is expected this will provide information to support clinical application and imaging diagnosis in the future.

## 2. Materials and Methods

### 2.1. Study Population

This experiment was a retrospective study. It collected the ^99m^Tc-TRODAT-1 imaging and diagnostic reports archived in the Picture Archiving and Communication System (PACS) between March 2006 and August 2013. A total of 202 cases were collected. Among these were six healthy patients (three males and three females) and 196 patients with PD (80 males and 116 females). These patients were between the ages of 25 and 91 (Table 2).

According to the disease stage, the number of healthy control (HC) and PD’s HYS stage I to V patients were 6, 22, 27, 53, 87, and 7, respectively. In the study, the category was distinguished into healthy, early (HYS I, HYS II), mid (HYS III), and four late groups (HYS IV, HYS V). Then, five slice images from each patient was chosen for the analysis data set. The total number of images was 1010 (healthy (n = 30), early (HYS I~II, n = 110, 135), mid (HYS III, n = 265), late (HYS IV~V, n = 435, 35).

**Figure 1:**
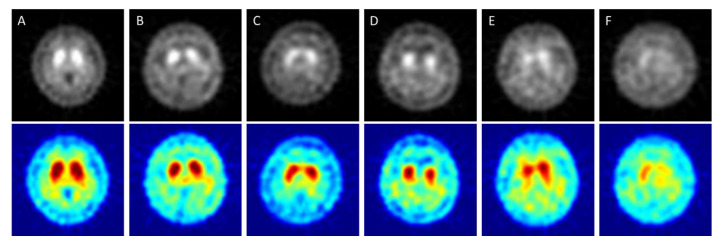
SPECT images correspond to HYS stage of PD ((**A**) = Healthy case, (**B**–**F**) = HYS I~V). The Upper row is original grayscale images. The lower images have pseudocolor mapping.

In Figure 1, “A” shows a healthy image. The white spot area showed high activity of striatum on both sides and had a curved bean shape. “B” corresponds to HYS I. The putamen in one-side of the striatum was decreased. “C” corresponds to HYS II. The putamen in two-sides of the striatum was decreased. “D” corresponded to HYS III. Two sides of the putamen show no activity. “E” corresponds to HYS IV. The two sides of the putamen display no activity and decreased activity on two sides of the caudate is seen. “F” corresponds to HYS V. There was no activity signal in the two sides of both the putamen and caudate.

Cases in which patients received an overall ^99m^Tc-TRODAT-1 dose of 25 to 30 millicuries (mCi) and then underwent imaging within 2.5 to 4 h after administration were included in the study. However, patients who experienced head tremors were excluded, along with patients who received drug treatment during imaging since the drugs’ interference with the efficacy of ^99m^Tc-TRODAT-1 would make outcomes unreliable. The Medical Ethics Committee of E-DA Hospital approved this clinical study (EMRP-100-054(RIII)). All patients signed an informed consent form prior to their participation.

### 2.2. The Imaging Conditions of ^99m^Tc TRODAT-1 SPECT

A step-by-step (10 min, 32 steps) scan method was adopted to perform a DaTscan using ^99m^Tc-TRODAT-1. The image matrix size was 128 × 128. A total of 64 images were captured at a collection rate of 25 s per image. Filtered back projection (FBP) was adopted for image reconstruction. The filter was a low-pass Butterworth filter with a cutoff frequency of 0.4 and an order of eight. A dual-head SPECT instrument (E. cam^TM^ Signature Series Fixed 180; Siemens Medical Solutions Inc., Malvern, PA, USA) equipped with the Siemens E. soft Workstation and a fan beam collimator was employed in this study. The field of view (FOV) of a single detection head was 53.3 × 38.7 cm^2^, and the diagonal FOV was 63.5 cm. A single detection head is equipped with 59 photomultiplier tubes in a hexagonal arrangement and uses 5/8 inch sodium iodide (NaI(Tl)) crystals with a crystal size of 59 × 44.5 cm^2^.

### 2.3. The Deep Learning Method Concept

Deep convolutional neural network technology has five primary layers which are named convolution layer, pooling layer, rectified linear unit (ReLU) layer, fully connected layers, and softmax layer. The above steps are performed to define the feature category that satisfies the input image. This study designed and established a PD staging classification model through the pre-trained network in the above manner. Moreover, it evaluated the model effectiveness with accuracy, recall, precision, F-score, and Kappa. NVIDIA GeForce GTX 1060 6 GB (Santa Clara, CA, USA) hardware was used to train the CNN in this study.

### 2.4. Popular Pre-trained Models in CNN

**Figure 2:**
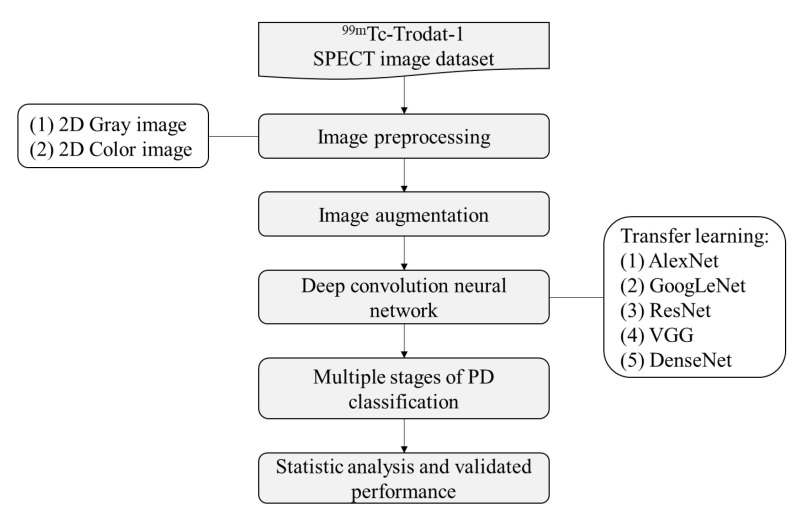
The study flow chart in multiple stages classification of Parkinson’s disease.

In this study, a total of 1010 DaTscan slice images was explored in the CNN analysis using five different pre-trained models. These were the AlexNet, GoogLeNet, Residual Neural Network, VGG, and DenseNet models. There were two types of image data sets: grayscale and pseudocolor images. For each subject only the maximum active slice of the striatum and two other slices, which were the previous and next one to the active slice were obtained in order to simulate how to diagnose and determine the disease in the clinic. Therefore, five images collected from each patient were used in the analysis dataset. The total number of images was 1010 (early (HYS I~II, n = 110, 135), mid (HYS III, n = 265), late (HYS IV~V, n = 435, 35) and healthy (n = 30)). Furthermore, 70% of the data was used for training and 30% of the data was used for verification. The overall structure of the study’s analytic system is illustrated in Figure 2. In Step 1, the ^99m^Tc-Trodat-1 SPECT images, which were already separated into six categories were input. In Step 2, these SPECT images were colored by the pseudocolor technique. The pseudocolor technique was associated with a color table that defines the color displayed for each image pixel. In Step 3, the image augmentation method was used to increase the number of images. In Step 4, we start to train the network using the five pre-trained models. Before the training process begins, each SPECT image needs to be resized to match every model’s input size. In Step 4, the multiple stages of the PD classify model were established. Finally, in Step 5, the testing set data was used for statistical analysis and performance validation.

#### 2.4.1. AlexNet

The architecture of the AlexNet network could be regarded as a large LeNet. The input image size was increased from 28 × 28 to 224 × 224 (RGB). It was divided into a five-layer convolution neural network in the front part and a traditional connection layer. There are a total of eight layers in AlexNet. The input of this architecture was an image and the output was the prediction classification and error rate. The AlexNet network improves the ReLU and pooling layers of the LeNet network by using ReLU to replace the previously used sigmoid and tanh functiond. It can solve the problem of gradient disappearance, make training more efficient and improving the accuracy. In the pooling layer, the LeNet network uses the average pooling method but the features will be blurred, whereas the AlexNet network uses the max-pooling method and chose a small step, which can avoid feature loss.

The AlexNet network uses two methods to reduce overfitting. First, a data augmentation technique [27] is used to increase the amount of training data to avoid overfitting during training, which is due to insufficient images in the training data set. The data augmentation technique includes image flip and horizontal and vertical moves to obtain a different view of the same image to provide more images for training. Second, the dropout method [28] is used to add a dropout in the fully connected layer. Each neuron with the same probability does not participate in the transfer of the next layer. This method makes the network force the current neurons for training that effectively reduce the overfitting. The dropout rate of the AlexNet was 0.5, which means each neuron had a 50% chance of not participating in the transmission of the next layer.

**Figure 3:**
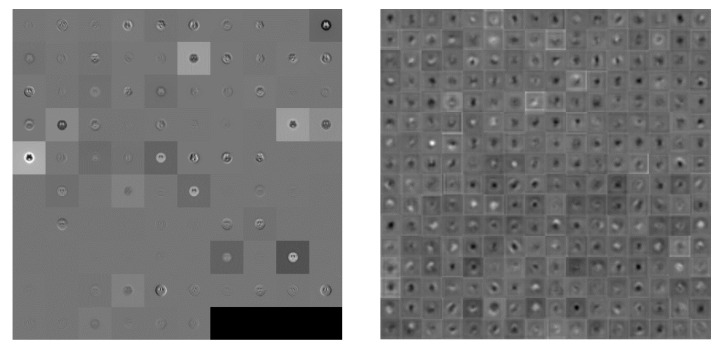
Visualization of the activation of the convolution layer in AlexNet (**Left**: first convolution, **Right**: final convolution).

This study utilized a pre-trained AlexNet model. The input image size was 227 × 227, using five convolution, three max-pooling, seven ReLU and two fully connected layers, followed by a softmax output layer (about the convolution parameter and pooling layer as shown in Table 3). A total of 25 layers were discussed for gray and color images in four and six categories simultaneously. Figure 3 shows visually the convolution between first and final image.

#### 2.4.2. GoogLeNet

GoogLeNet first appeared in ILSVRC14 competition and was the champion with a top-5 error of 6.67% [29]. GoogLeNet has a bold structure, with 22 layers in depth. Nevertheless, the parameter number was much smaller than in AlexNet. GoogLeNet has five million parameters and AlexNet has 60 million parameters, or twelve times more that GoogLeNet. Therefore, GoogLeNet is a better choice when memory or computing resources are limited.

Generally, the best way to improve network performance ss to increase the network’s depth and width. Depth refers to the number of network layers and width refers to the number of neurons. However, this method had three problems: (1) If the training data set is not enough and has too many training parameters, there will be overfitting. (2) If we design more layers to be included in the network, more parameters will be needed and the computation will be more complicated. (3) If the network becomes deeper, it will produce a gradient disappearance problem. To solve these problems, the solution is to reduce the parameters while increasing the depth and width of the network.

Google proposed the original inception module structure. The structure uses three convolution layers (1 × 1, 3 × 3, 5 × 5) and one pooling layer (using max pooling) for stacking to increase the network’s width. A ReLU function was required after each convolution layer to increase the non-linear characteristics. However, in the original inception version, the calculation for a 5 × 5 convolution kernel was too large, which caused the feature map dimensions to be too big. To avoid this situation, a 1 × 1 convolution kernel is added before the 3 × 3, 5 × 5 convolutions and after max pooling was produced. This could successfully reduce the feature map dimensions, increase the non-linear characteristics of the network and improve the expressive ability of the network. For example, if the output size of the previous layer was 28 × 28 × 192, after passing through 5 × 5 convolution with 32 channels the output size will become 28 × 28 × 32. Moreover, the parameters of the convolution layer were 192 × 5 × 5 × 32. After passing through the first 64 channels by 1 × 1 convolution and then 32 channels 5 × 5 convolution the output size was still 28 × 28 × 32, but the convolution parameters are reduced to (192 × 1 × 1 × 64) + (64 × 5 × 5 × 32). This is a significant reduction of the number of parameters.

**Figure 4:**
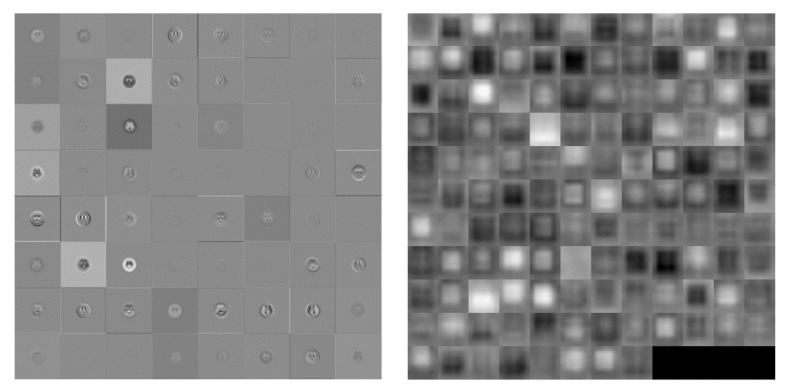
Visualization of the activation of the convolution layer in GoogLeNet (**Left**: first convolution, **Right**: final convolution).

This study utilized a pre-trained GoogLeNet model. The input image size was 224 × 224, using 58 convolutions (in the inception module it had 55 convolutions), 14 max-pooling layers (the inception module had nine pooling layers), and one layer fully connected by a softmax output layer. A total of 144 layers was used for gray and color images in four and six categories simultaneously. Visually the difference between the first and final convolution is shown in Figure 4.

#### 2.4.3. ResNet 

**Figure 5:**
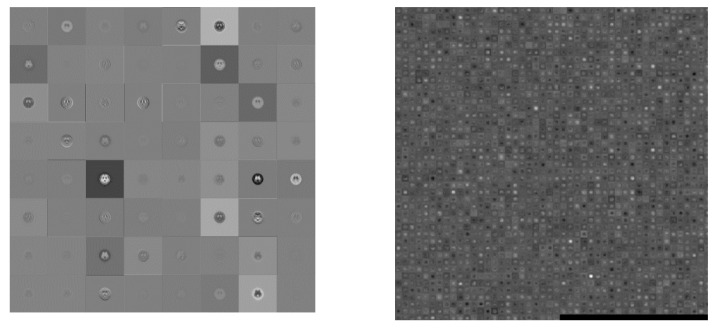
Visualization of the activation of the convolution layer in ResNet50 (**Left**: first convolution, **Right**: final convolution).

**Figure 6:**
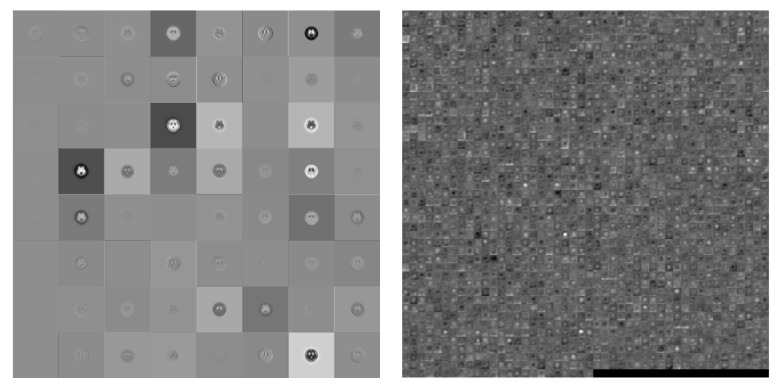
Visualization of the activation of the convolution layer in ResNet101 (**Left**: first convolution, **Right**: final convolution).

In the ILSVRC15 competition, the residual neural network (ResNet) technique won the championship with a top-5 error of 3.57% by using depth layers 152 [30]. Its characteristic was that the neural network did not necessarily need to be executed layer by layer. It could skip to the next layer through jumping. For a CNN, it is very important to identify the depth, but a deeper network will result in more complexity. The common reason is that the backpropagation is hard to update when the network is too deep. At the same time, this will cause the training speed to increase. Besides, it is found that the deep network causes degradation problems. This study utilized a pre-trained ResNet50 model. The input image size was 224 × 224 ResNet50 and ResNet101. (1) ResNet50: using 53 convolution, one max-pooling, and one fully connected layer, followed by a softmax output layer. A total of 117 layers was used for gray and color images in four and six categories simultaneously. The difference between the first and final convolution is visualized in Figure 5 and Figure 6.

#### 2.4.4. VGG

VGG is the abbreviation for the Visual Geometry Group of Oxford University in the United Kingdom. Its main contribution is the use of more hidden layers and more training images, which can improve the accuracy to 90%. The VGG network can be divided into VGG16 and VGG19, which have 16 layers (13 convolution layers and three full connection layers) and 19 layers (16 convolution layers and three full connection layers), respectively [31]. The architecture is described below.

Compared with AlexNet, VGG16 uses several consecutive 3 × 3 convolutions to replace the larger convolutions (11 × 11, 7 × 7, 5 × 5) of the former. Given a local size picture as input multiple small convolutions are used because multiple non-linear layers can increase the network’s depth, which not only ensures the complexity of learning but spends fewer resources. In VGG, three 3 × 3 convolutions were used to substitute for a 7 × 7 convolution, and two 3 × 3 convolutions were used to substitute for a 5 × 5 convolution. The purpose was to ensure that the depth and effect of the network were improved under the same conditions. For example, three 3 × 3 convolutions with a step equal to 1 can be regarded as an input image with a 7 × 7 size in layer operation. The total number of parameters was 27 × C^2^ (C is the number of input and output channels). Suppose we use a 7 × 7 convolution kernel, then the total number of parameters is 49 × C^2^. The former not only reduces the parameters but also better maintains the image properties.

The main advantage of VGG is that its structure is straightforward. The entire network uses the same size of convolution (3 × 3) and maximum pooling size (2 × 2). The combination of several small convolution layers (3 × 3) was better than one large convolution layer (5 × 5, 7 × 7). It was verified that the continuously deep network structure could improve the performance. The disadvantage of VGG is that it consumes more computing resources and uses more memory. Most of the parameters are in the fully connected layer, and VGG has three fully connected layers.

**Figure 7:**
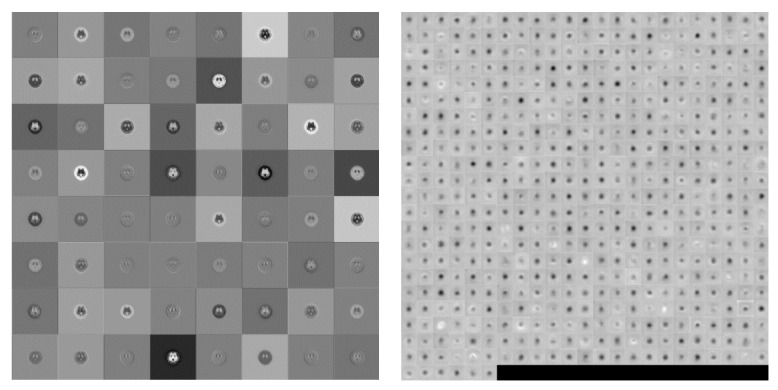
Visualization of the activation of the convolution layer in VGG19 (**Left**: first convolution, **Right**: final convolution)

This study utilized the VGG19 pre-trained model. The input image size was 224 × 224, using 16 convolution, five max-pooling, and three fully connected layers, followed by a softmax output layer. A total of 47 layers was used for the gray and color images in four and six categories simultaneously. In visualize the result the difference between the first and final convolution is shown in Figure 7.

#### 2.4.5. DenseNet

**Figure 8:**
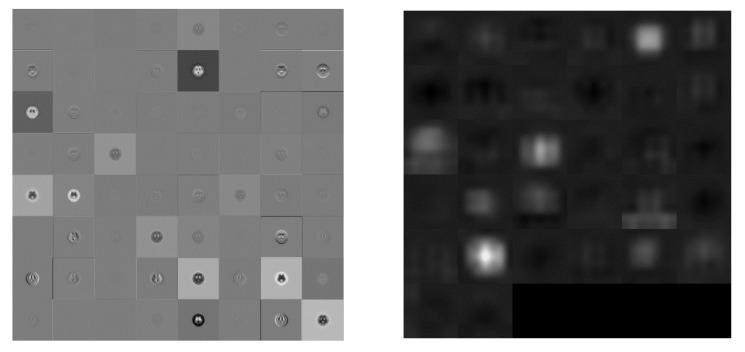
Visualization of the activation of the convolution layer in DenseNet201 (**Left**: first convolution, **Right**: final convolution).

DenseNet is similar to ResNet. The difference between DenseNet and ResNet is that ResNet calculates summations, but DenseNet calculates by stitching. The input of each layer network includes the previous output. For example, the L^th^ layer’s input is equal to *k0+k(L-1)*, where *k* is the number of channels. DenseNet improves the transmission efficiency of information and gradients in the network. Each layer can directly get the gradient from the loss function and get the input signal directly. In this way, the network can be trained deeper. This structure also has the effect of regularization. Relatively, other networks are committed to improving network performance based on the depth and width [32]. The characteristic of DenseNet is that the features trained in each layer are provided repeatedly for use in subsequent layers. This greatly improves the feature utilization rate. The advantages of DenseNet are: (1) It alleviates the vanishing-gradient problem. (2) It strengthens the spread and reuse of features. (3) It significantly reduces the number of parameters. This study utilized the DenseNet201 pre-trained model. The input image size was 224 × 224, 200 convolutions, one max-pooling, and one fully connected layer, followed by a softmax output layer. A total of 709 layers was used for the gray and color images in four and six categories simultaneously. The difference between a first and final convolution is visualized in Figure 8.

This study discusses five commonly used pre-trained models for Parkinson’s disease multistage classification. Table 4 lists the parameters of these six models, including the input image size, depth, layer number, model memory size, and training time based on a batch size of 10 when the epoch was 1.

## 3. Experimental Results

**Figure 9:**
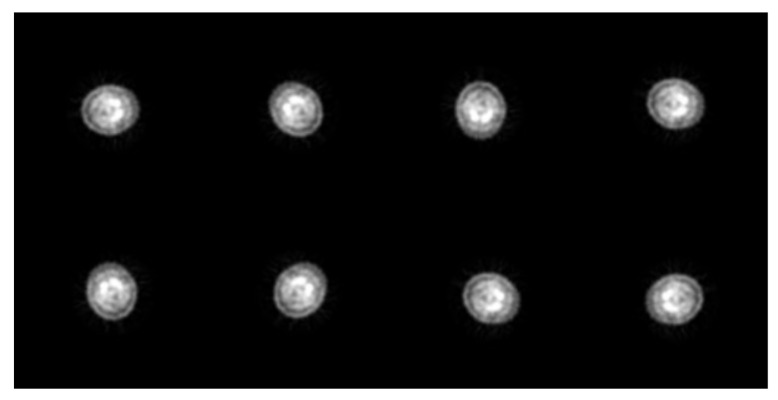
Example of image augmentation by reflection, rotation (−180, 180), *X*-axis translation (−3, 30), *Y*-axis translation (−3, 3).

This study was utilized five pre-trained models—AlexNet, GoogLeNet, Residual Neural Network, VGG, and DenseNet—to classify Parkinson’s disease stages. A total of 202 DaTscan cases were collected in this study. They are separated into two groups. One is healthy, early, mid, and late PD (four categories). The other is healthy and HYS I~V (six categories). There were two kinds of images in the deep CNN training data sets: grayscale and pseudocolor images in order to simulate the clinical diagnosis and determine the disease stage. Only the striatum’s maximum active slice and the active slice’s previous and next two images were obtained for each subject. Therefore, the analysis data set contains five images collected from each patient. The total number of images was 1010 (early (HYS I~II, n = 110, 135), mid (HYS III, n = 265), late (HYS IV~V, n = 435, 35), healthy (n = 30), and then 70% of the data in the data set used for training and 30% of the data used for verification. Due to the fact the number of images in each category was unbalanced an imported image augmentation method (such as cropping, rotating, resizing, translating, and flipping) was applied (Figure 9).

This study uses recall, precision, F-score, accuracy, and Kappa to evaluate the performance of the classification models. Recall means the percentage of positive predictions in the total of positive cases. Precision means how many true positive cases are in the total number of positive predictions. When the recall is low, it may not be possible to judge the category, but it will not be misjudged if the model provides a result. When the precision is low, it means the category cannot be determined correctly. For both evaluation indexed bigger is better. If a result has high precision and low recall, it means the model might be too cautious and almost useless for prediction. If a result has high recall and low precision it means the model might produce more misjudgment results. F-score is the harmonic means between recall and precision. One can utilize this index to roughly evaluate model performance. Accuracy is the total classification performance of the model. Kappa is used to evaluate the agreement of the classification results compared with real cases.

Table 5 shows each pre-trained models’ deep CNN results divided between grayscale and color images in four categories: healthy, early, mid, and late PD. As can be seen from the data AlexNet had the best performance on the grayscale images with accuracy, recall, precision, F-score, and Kappa values of 0.825, 0.753, 0.874, 0.809, and 0.725, respectively, while DenseNet201 had the best performance on the color images with accuracy, recall, precision, F-score, and Kappa values of 0.855, 0.821, 0.903, 0.860, 0.724, respectively.

Table 6 shows the deep CNN results of each pre-trained models between grayscale and color images in four categories: healthy and Parkinson disease stages I to V. AlexNet had the best performance on the grayscale image with accuracy, recall, precision, F-score, and Kappa values of 0.774, 0.742, 0.853, 0.794, and 0.679, respectively, whereas DenseNet201 had the best performance on the color images with accuracy, recall, precision, F-score, and Kappa values of 0.778, 0.696, 0.814, 0.750, 0.680, respectively.

**Figure 10:**
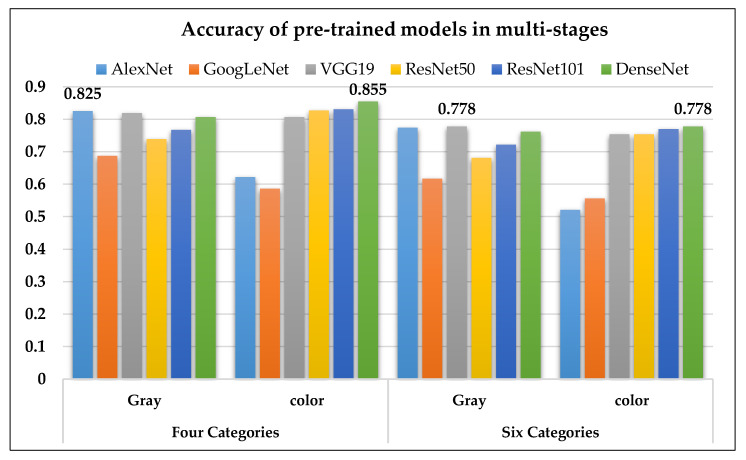
The accuracy of the six pre-trained models on four and six stages of PD1.

In terms of accuracy, the six pre-trained models were divided into four and six stages for PD. The classification performance using grayscale images was best with AlexNet (four stages) and VGG19 (six stages). In addition, the classification performance using pseudocolor images was best with DenseNet for both the four and six stages (Figure 10).

## 4. Discussion

Nuclear medical imaging is a kind of functional imaging. The images mainly show the intensity of the radiopharmaceutical activity of the organ. In this study’s deep learning design, using a pre-trained model could reduce the time spent on the CNN model development, but the use of the pre-trained model needs to conform to the model architecture. Furthermore, transfer learning needs to be carried out before training. Two points in the model must be revised: (1) The image size of the source data needs to be adjusted and (2) The number of output categories needs to be corrected. Besides, it can obtain a better model during training by changing parameters such as batch size, epoch and learning rate. Batch size refers to how many data pieces were used to calculate each iteration, and epoch represents the training sample run repeatedly.

### 4.1. Comparison between Published Literature Methods and the Presented Method

PD patients are commonly into two normal and abnormal groups (Table 7). There are fewer articles that discuss multi-class classification. Therefore, this study tried to use two kinds of techniques to explore multi-class classification. Finally, we successfully found a CNN model to classify the healthy and HYS I~V stages of PD diseases and obtained high accuracy. However, with the CNN model it was more difficult to explain the characteristics adopted by the model and the correlation between the characteristics and PD disease stages.

### 4.2. The Related Literature on Multiple Stages Classification in Medical Images

Regarding the classification of multi-stage diseases in medical images, Farooq et al. published in 2017 an article titled, “*A deep CNN-based multi-class classification of Alzheimer’s disease using MRI*” [33]. The authors divided Alzheimer’s disease into four groups: Alzheimer’s, mild cognitive impairment, late mild cognitive impairment and healthy persons. Then by jusing a pre-trained model, they successfully achieved a high classification accuracy rate of 98.8%.

In 2019, Talo et al. published the article “*Convolutional neural networks for multi-class brain disease detection using MRI images*” [34], which classified five class of MRI images of the brain, namely normal and cerebrovascular, neoplastic, degenerative and inflammatory diseases categories. It also used a pre-trained model and obtained 95.23% classification accuracy. In 2020, Ramzan et al. published “*A Deep Learning Approach for Automated Diagnosis and Multi-Class Classification of Alzheimer’s Disease Stages Using Resting-State fMRI and Residual Neural Networks*” [35]. It divided the disease into six categories: cognitively normal, significant memory concern, early mild cognitive impairment, mild cognitive impairment, late mild cognitive impairment, and Alzheimer’s disease. Moreover, using the pre-trained model plus transfer learning they obtained the accuracy of 97.92%. At the same time, the conclusiond mentioned that the pre-trained model could be used for multi-stage classification, which is consistent with this study’s conclusion.

### 4.3. The Presented Deep Learning Method

The images collected for this paper was a DICOM image file after DaTscan. There are two approaches in the literature on deep learning. One is to select a slice image of the striatum for modeling. The other one is to use the entire group of brain images for modeling. This study combines the advantages of both the above methods to design and select a striatum slice and choose the other two slices before and after the striatum. Then we assemble these five slices as an image data set. This can increase the amount of data in the image set and eliminate the noise of the external striatum. This method also corresponds to how clinicians perform interpretation and disease diagnosis. Many studies have pointed out that more training data could provide higher accuracy and stability [36]. However, more data takes more time to train the model and hardware equipment support is required. If the hardware performance is low the training time will be longer. It will be impossible for model training and modeling (due to problems like insufficient memory) in some cases (as shown in Table 8).

## 5. Conclusions

The assessment of pathophysiological changes using SPECT imaging could be essential for the diagnosis of Parkinson’s disease. The purpose of this study was to classify the multiple stages of PD by using pre-trained deep CNN models. In deep learning, the five CNN models Alex Net, GoogLeNet, Residual Neural Network, VGG, and DenseNet were used for modeling and to determine the accuracy of distinguishing four and six categories in PD staging. When performing deep learning, there were a total of 829 PD images. 70% of the data was used as the training set and 30% of the data was used for verification. The original grayscale image and the pseudo color technique to produce color images were compared with the classification results. The following is a summary of the conclusions obtained in this study.

- When using deep convolutional neural network technology to classify ^99m^Tc-Trodat-1 PD images for the original grayscale images processed through five pre-trained models (AlexNet, GoogLeNet, VGG19, ResNet, DenseNet201) the highest accuracy was 0.83 for AlexNet. In six categories (healthy, HYS I~V), the best accuracy was 0.78 obtained by VGG19 in four categories (healthy, early, mid, late);
- For color images, DenseNet201 yielded the highest accuracy of 0.85 in four categories. In six categories, the highest accuracy was 0.78 also obtained using DenseNet201;
- Overall, the pre-trained models could produce accurate results when using grayscale images. In this case, the pseudocolor images might be non-essential;
- CNN could obtain high classification accuracy in multiple categories of SPECT PD scans;
- However, the establishment of the CNN classification model was very time-consuming, and the results had low interpretability in clinic.

## Figures and Tables

**Table 1:** The Hoehn and Yahr scale and symptoms.

Stage	Hoehn and Yahr Scale
1	Unilateral involvement only usually with minimal or no functional disability.
2	Bilateral or midline involvement without impairment of balance.
3	Bilateral disease: mild to the moderate disability with impaired postural reflexes; physically independent.
4	Severely disabling disease; still able to walk or stand unassisted.
5	Confinement to bed or wheelchair unless aided.

**Table 2:** Demographic information of the dataset.

Class	Subjects	Sex	Age	Slice Images
HC	6	3 F, 3 M	48 ± 14.7	30
HYS I	22	13 F, 9 M	68 ± 16.4	110
HYS II	27	15 F, 12 M	69 ± 10.3	135
HYS III	53	36 F, 17 M	71 ± 9.8	265
HYS IV	87	47 F, 40 M	69 ± 10	435
HYS V	7	5 F, 2 M	65 ± 11.5	35

**Table 3:** The parameter of convolution and pooling layer in AlexNet.

Item	Conv1	Pool1	Conv2	Pool2	Conv3	Conv4	Conv5	Pool5
Filter size	11 × 11	3 × 3	5 × 5	3 × 3	3 × 3	3 × 3	3 × 3	3 × 3
Stride	4	2	1	2	1	1	1	2
Padding	0	0	2	0	1	1	1	0

**Table 4:** Pre-trained models comparison. Experimental parameters: Batch size = 10; Epoch = 1

Name	Input Size	Layers	File Size	Training Time(s)
AlexNet	227 × 227	25	227 MB	25.4
GoogLeNet	224 × 224	144	27 MB	62.7
ResNet50	224 × 224	114	96 MB	138.4
ResNet101	224 × 224	347	167 MB	326.1
VGG19	224 × 224	47	535 MB	162.1
DenseNet201	224 × 224	709	77 MB	880.7

**Table 5:** The pre-trained model performance in four categories on the difference image format.

Category = 4 (Healthy, Early, Mid, and Late PD)								
Net	Image Format	Batchsize	MaxEpochs	Accuracy	Recall	Precision	F-Score	Kappa
AlexNet	Gray	80	40	0.825	0.753	0.874	0.809	0.725
	Color	162	81	0.622	0.504	0.701	0.587	0.397
GoogLeNet	Gray	14	7	0.687	0.673	0.728	0.700	0.518
	Color	34	17	0.586	0.492	0.672	0.568	0.368
VGG19	Gray	20	10	0.819	0.758	0.870	0.810	0.720
	Color	24	12	0.807	0.808	0.838	0.823	0.707
ResNet50	Gray	8	4	0.739	0.729	0.710	0.719	0.607
	Color	8	4	0.827	0.837	0.749	0.791	0.743
ResNet101	Gray	6	3	0.767	0.691	0.668	0.679	0.656
	Color	12	6	0.831	0.824	0.857	0.840	0.744
DenseNet	Gray	18	9	0.807	0.722	0.843	0.778	0.704
	Color	16	8	0.855	0.821	0.903	0.860	0.724

**Table 6:** The pre-trained models performance in six categories on the difference image format.

Category = 6 (Healthy and Parkinson Disease Stages I to V.)								
Net	Image Format	Batchsize	MaxEpochs	Accuracy	Recall	Precision	F-Score	Kappa
AlexNet	Gray	96	48	0.774	0.742	0.853	0.794	0.679
	Color	120	60	0.521	0.349	0.491	0.408	0.282
GoogLeNet	Gray	34	17	0.617	0.532	0.695	0.603	0.439
	Color	28	14	0.556	0.378	0.478	0.422	0.362
VGG19	Gray	24	12	0.778	0.612	0.665	0.637	0.683
	Color	22	11	0.754	0.746	0.747	0.747	0.669
ResNet50	Gray	20	10	0.681	0.56	0.768	0.647	0.537
	Color	18	9	0.754	0.661	0.758	0.706	0.651
ResNet101	Gray	16	8	0.722	0.602	0.645	0.623	0.603
	Color	18	9	0.770	0.699	0.777	0.736	0.673
DenseNet201	Gray	16	8	0.762	0.670	0.739	0.703	0.661
	Color	8	4	0.778	0.696	0.814	0.750	0.680

**Table 7:** Comparison with related works of PD classification.

Author (year) [reference]	Category	Sample Size	Method/# Feature	Classifier	Accuracy
R. Prashanth et al. (2016) [37]	Normal	208	Machine learning/34	SVM	0.97
	PD	427			
Abdelbasset Brahim et al. (2017) [38]	Normal	111	Voxels as Features approach and Principal Component Analysis	SVM	0.88
	PD	158			
Ehsan Adeli et al. (2017) [39]	Normal	169	Kernel-based Feature	SVM	0.95
	PD	369			
Mosarrat Rumman et al. (2018) [40]	Normal	100	ROI detection and area calculation	ANN	0.94
	PD	100			
Presented Method: Deep CNN	4 stage	Healthy = 6 early = 245 mid = 265 late = 470	Popular Pre-trained models in CNN	Alex Net (grayscale)	0.83
				DenseNet (color)	0.85
	6 stage	Healthy =6 HYS1= 110 HYS2 = 135 HYS3 = 265 HYS4 = 435 HYS5 = 35	Popular Pre-trained models in CNN	VGG19 (grayscale)	0.78
				DenseNet (color)	0.78

**Table 8:** Two sample sizes for PD stage classification demonstrated in VGG19 (hardware environment: NVIDIA GeForce GTX 1060 6GB).

Images	Batch Size	Epoch	Training Time (s)	Accuracy
327	10	10	602.87	0.55
327	20	10	2942.46	0.56
327	30	10	3905.14	0.54
327	40	10	GPU out of memory	
672	10	10	236.36	0.78
672	20	10	4537.95	0.77
672	30	10	8318.34	0.63
672	40	10	GPU out of memory	
